# Rapid Anti-tTG-IgA Screening Test for Early Diagnosis of Celiac Disease in Pediatric Populations

**DOI:** 10.3390/nu15234926

**Published:** 2023-11-26

**Authors:** Irati Mendia, Verónica Segura, Ángela Ruiz-Carnicer, Laura Coto, María Negrete, Joshua C. D. Long, Joaquin Reyes, Benito Amil, Ignacio Salamanca, Isabel Comino, Ángel Cebolla, Carolina Sousa

**Affiliations:** 1 Biomedal S.L., 41900 Seville, Spain; irati.mendia@biomedal.com (I.M.);; 2 Department of Microbiology and Parasitology, Faculty of Pharmacy, University of Seville, 41012 Seville, Spain; 3 Instituto Hispalense de Pediatría, 41014 Seville, Spainignaciosalamanca@ihppediatria.com (I.S.)

**Keywords:** celiac disease diagnosis, gluten intake, anti-tTG-IgA, PoCT, LFIA

## Abstract

A large number of patients with celiac disease (CD) remain undiagnosed because they do not fulfill the criteria for entry into the conventional diagnostic workflow. This study evaluated the clinical utility of anti-tissue transglutaminase IgA antibody lateral flow immunoassays (anti-tTG-IgA LFIA) in the undiagnosed-CD-based pediatric population and the impact of a gluten-free diet (GFD) on screening-detected CD. A total of 576 volunteers were tested for anti-tTG-IgA. Gluten consumption habits, CD related symptoms, and risk factors for CD development were evaluated. Volunteers testing positive for anti-tTG-IgA were referred to the conventional CD diagnostic workflow, and the impact of the GFD on health-related quality of life (HR-QoL) was measured. Among them, 13 had a positive anti-tTG-IgA LFIA test result: 11 had confirmed CD (1.91%), one refused confirmatory tests, and another is undergoing diagnosis. Regarding the CD prevalence, no significant differences were observed among risk (1.89%) and symptomatic (2.65%) groups and the entire tested population (1.55%). Rapid anti-tTG-IgA LFIAs could be of clinical utility in primary care for the early identification of children with CD unidentified by the conventional diagnostic workflow. It could potentially reduce the costs of undiagnosed CD, avoiding unnecessary referrals to gastroenterologists, reducing diagnosis delays and long-term problems, and improving patients’ HR-QoL.

## 1. Introduction

Celiac disease (CD) is a lifelong chronic immune-mediated systemic disorder triggered by dietary gluten exposure in genetically susceptible individuals [1,2]. Currently, 45–90% of affected patients remain undiagnosed, constituting a public health problem worldwide; likewise, diagnostic delays can range from months to more than 10 years [3,4,5,6,7,8,9,10,11,12,13]. The variability in the percentage of undiagnosed CD is multifactorial. Country awareness about CD, in both the clinician and population side, access to confirmatory tests, socioeconomic status, self-perceived health status, sex, and age are some of the variables that modify this percentage [3,4,6,8,9,10,11,12,13]. CD is conventionally diagnosed using a combination of serology and duodenal biopsy, with the detection of anti-tissue transglutaminase IgA antibodies (anti-tTG-IgA) and total IgA antibodies recommended as the first-line test [14,15]. Some of the most recent guidelines, such as the European Society for Paediatric Gastroenterology Hepatology and Nutrition (ESPGHAN) 2020 ones, have incorporated a no-biopsy approach as the first pathway for diagnosing CD in children, which allows the direct confirmation of CD in children whose anti-tTG-IgA titers are more than 10 times (10×) the upper limit of normal (ULN) and anti-endomysial antibodies (anti-EMA) are positive [14,15]. Using this approach, 50–75% of patients can be diagnosed without further tests, resulting in a decrease in costs, risks, and refusals of diagnosis [14,16,17]. However, it is important to be aware that to avoid false-negative results complementary tests must be performed, and those who are tested need to maintain a gluten-containing diet to achieve active CD [1,2,18].

There are three principal options for identifying potential patients with CD: active case-finding, risk-group screening, and mass screening. For active case-finding, physicians must be aware of the broad range of CD manifestations to direct patients for CD testing in a timely manner. The clinical phenotypes of CD are termed “gastrointestinal” (GI) or “extraintestinal” (EI) depending on the symptom’s location, which may occur either individually or in combination [1,2]. Among common GI symptoms are chronic diarrhea, steatorrhea, abdominal pain, bloating, constipation, heartburn, recurrent vomiting, or nausea, whereas for EI symptoms can be manifested almost in every organ in the body; as CD resembles a multisystem disorder, it may appear in, among others, dermal, oral, cardiovascular, musculoskeletal, mental health, pulmonary, renal, liver, endocrine, reproductive, or hematologic conditions [1,2]. The wide variety of symptoms, together with their potential variation over time, complicates active case-finding. Furthermore, it is important to consider that two-thirds of the patients with CD have symptoms which are below the threshold of clinical detection (asymptomatic patients) [3,8,19]. Together, these aspects result in a low predictive value for symptom based active case-finding [20].

The clinical value of mass screening is a debated topic [14,19,21,22]. CD meets most of the World Health Organization criteria for disease screening because it is a common, detectable, and treatable disease that can lead to complications when undetected [23]. Opponents advocate for screening only at-risk populations, such as in first-degree relatives of patients with CD (CD-FDR) or patients with certain autoimmune disorders or genetic based syndromes, among which the CD prevalence is approximately 5–10%, compared to 1.4% (95% confidence interval (95%CI) 1.1–1.7%) in the general population [7,24,25,26,27].

However, it is important to consider that the clinical picture of CD is continuously evolving, with changes in prevalence, improvements in diagnostic methods, increased understanding of pathology, and improved options to facilitate a gluten-free diet (GFD), leading to changes in the value of screening approaches. For example, the prevalence of CD seems to have increased decennially, since there have been records, and may first appear at any age [7,13,28,29,30]. Further, patients with CD diagnosed during childhood have been shown to achieve higher adherence to treatment, better GI mucosal recovery, reduced long-term severe complications and associated therapeutic treatments, and reduced strain on health systems [8,28,31,32,33,34,35,36,37,38,39]. 

This study aimed to investigate the clinical utility in primary care of rapid anti-tTG-IgA lateral flow immunoassays (LFIA) in CD screening of 2- to 14-year-olds, included both general and high-risk populations. The clinical utility of anti-tTG-IgA LFIA was evaluated by determining the positive predictive value (PPV) of the tool and the frequency of undiagnosed patients with CD in these populations. Additionally, test results were categorized according to test line intensity in order to establish a relationship between saturated test lines and anti-tTG-IgA concentration above 10× ULN (one of the main criteria for no-biopsy diagnosis approach in children), and unsaturated test lines and a positive ELISA result but below 10× ULN, a perspective which to our knowledge has not been published before [14,15]. Furthermore, the health-related quality of life (HR-QoL) outcomes of the volunteers diagnosed were analyzed to test the impact of screening on their health.

## 2. Materials and Methods

### 2.1. Study Design and Volunteers

A cross-sectional observational study of CD screening was conducted at 12 different centers in Seville and Madrid (Spain) from November 2021 to March 2023. The study was conducted according to the guidelines of the Declaration of Helsinki and approved by the Ethics Committee of Hospital Universitario Virgen Macarena (Seville, Spain) (n. 2021/273) (ClinicalTrials.gov identifier NCT05186038). Informed consent was obtained from all parents or legal guardians of subjects involved in the study, as they were under 14 years old.

**Figure 1:**
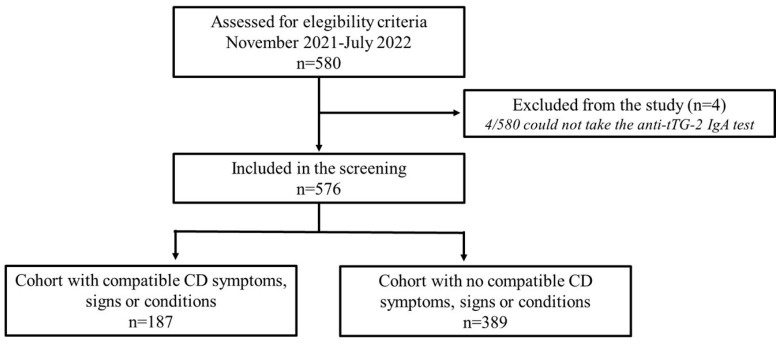
Flowchart of the participants.

Once the written consent was obtained, the volunteers were in situ tested for whole blood anti-tTG-IgA LFIAs in order to detect CD. In addition, volunteers were asked about their gluten consumption habits and completed a clinical questionnaire on symptoms related to CD and other risk conditions for developing CD according to the ESPGHAN 2020 guidelines [14]. Further, the volunteers (the parents or legal guardians for children aged 2–12; volunteers themselves for those aged 13–14) were asked if they had a CD-FDR (to choose between yes/no; if yes, the relation had to be specified). They were also asked if they suffered from another autoimmune condition or had a certain genetic disorder for which CD was prevalent (to choose from among eight types), and to list their recurrent symptoms (to choose from among 14 types, if affirmative; severity and onset date to be specified) (Figure 1).

Volunteers with anti-tTG-IgA positive test result were scheduled to visit a pediatric gastroenterologist to confirm the presence of CD. Furthermore, to evaluate the change in HR-QoL of diagnosed CD volunteers, the EuroQol EQ-5D-Y life quality questionnaire (validated for Spain) was administrated to all the volunteers who tested positive at the time of screening, and those diagnosed with CD during the study also >6 months after having initiated the GFD [40,41]. All the questionnaires, gluten consumption habits, and clinical and the EuroQol EQ-5D-Y life quality were completed with the help of the professionals conducting the study.

### 2.2. Gluten Consumption Habits

As recurrent gluten exposure is critical for elevating the anti-tTG-IgA titers to detectable levels, the volunteers were asked about their gluten consumption habits which mainly aimed to identify any bias in gluten intake customs. This questionnaire aimed to determine how often (daily, occasionally, or never, occasionally being less than twice a week) volunteers ate products that are typically made with gluten. Volunteers were provided with a list of foods that are made from gluten-containing ingredients, such as bread, cookies, breakfast cereals, bakery products, pasta, or couscous, in order to facilitate their response.

### 2.3. Anti-tTG-IgA LFIA in Whole Blood

The determination of anti-tTG-IgA in whole blood was performed using a commercial LFIA (Celiac Detect, Biomedal S.L., Seville, Spain) according to the manufacturer’s protocol. After massaging and cleaning a fingertip with an alcohol pad, a puncture was made through an automatic lancet. The blood was collected through a glass capillary tube, with which blood was collected by capillary action by pressing on the drop. Once this 2 cm long capillary was full, it was inserted into the buffer bottle, and it was mixed by inversion. Five drops of this mixture were added to the LFIA. A visual reading was taken 10 min later. No strip was read beyond 15 min, due to the fact that false positive results may appear after this time. No extra material was needed to perform the test because the product contained all the necessary components. However, since the test was carried out on others, gloves and lab coats were used. The test’s lower limit of detection was 5 U/mL. Although the test was validated for qualitative use, the results were specified as weak, intermediate, or strong intensities to identify potential correlations with ESPGHAN anti-tTG-IgA titer parameters.

### 2.4. Pediatric Determination

The ESPGHAN 2020 guidelines for CD diagnosis were followed when volunteers showed a positive anti-tTG-IgA LFIA result and were immediately referred to a pediatric gastroenterologist [14]. The volunteers were further examined for the presence of symptoms, anti-tTG-IgA, anti-EMA and/or anti-deaminated gliadin peptide antibodies, HLA haplotypes, among others, and the need for an intestinal biopsy, depending on the physician’s criteria.

### 2.5. Monitoring the Evolution of HR-QoL and Symptoms

The volunteers diagnosed with CD were contacted for a follow-up appointment >6 months after diagnosis. As in the initial screening, the volunteers’ parents or legal guardians completed the Spanish version of the EuroQoL group EQ-5D-Y questionnaire that consists of the EQ-5D-Y descriptive system and the EQ VAS score [40,41]. The same person answered the questionnaire on both occasions. Using the descriptive EQ-5D-Y, the five dimensions of HR-QoL were measured: mobility, ability to look after oneself (self-care), ability to perform daily activities, presence of pain or discomfort, and presence of emotional difficulties (feeling worried, sad, or unhappy). The response options are “no”, “some”, or “severe current problems”. The EQ-VAS records respondents’ overall current health on a vertical visual analogue scale from 0 to 100, corresponding to their worst and best possible imagined health, respectively. To check the evolution of symptoms, all diagnosed volunteers were asked for CD-related symptoms as per the ESPGHAN 2020 guidelines [14].

### 2.6. Statistics

The data were recorded in a Microsoft Excel spreadsheet (version 2303, Microsoft, Washington, DC, USA). Numerical variables were presented as percentage means and 95% CI or medians with interquartile ranges (IQR). Dichotomous variables were compared using the Chi-square test or Fischer’s exact test, and the Mann–Whitney U test was used for the quantitative data. All other statistical methods were used as appropriate. Statistical analyses were performed using IBM SPSS Statistics for Windows (version 25.0; IBM Corp., Armonk, NY, USA). A *p*-value < 0.05 was considered significant.

## 3. Results

### 3.1. Subjects

The study population consisted of 580 child volunteers between 2–14 years of age. Four potential volunteers were excluded because their blood samples could not be obtained (Figure 1). Of this population, 313 (54.3%) were males and 263 (45.7%) females, with a median age of 8 years (IQR 4–10 years). The calls for screening were made orally, via email, through social networks, and with conventional media. 

The study was conducted in twelve centers, and the approach used for volunteer recruitment in each of them varied. Most participating centers (10/12) in this study were shelters, sports clubs, and camps, and included a primary care pediatric center. In the case of shelters, direct contact was made with the center coordinator, who scheduled a meeting with the guardians of the children. For sports clubs and camps, contact was made with the director, who sent an email and/or informational sheets to the legal guardians of enrolled children (aged 2 to 14 years). Regarding the primary care pediatric center, pediatricians referred children who primarily attended well-child check-ups to the study. Of the twelve participating centers, only two, the Celiac and Gluten-Sensitive Association of the Community of Madrid and the Celiac Association of Seville, had recruited children from a risk group making calls for enrollment through social media.

### 3.2. Clinical Evaluation

**Figure 2:**
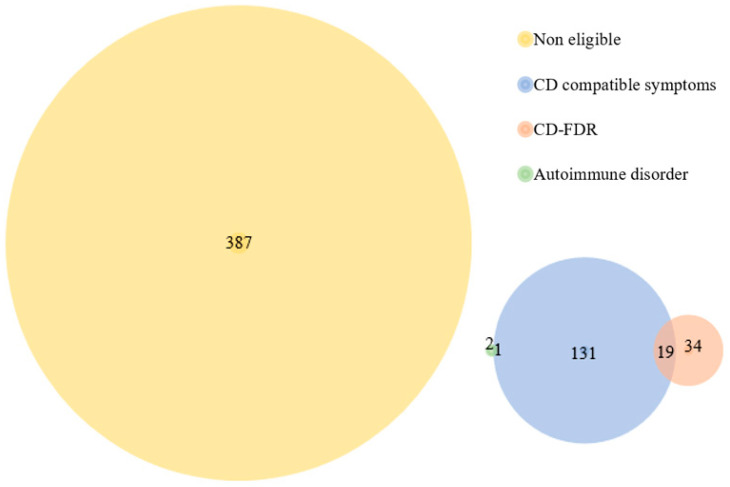
Venn diagram classifying volunteers according to their eligibility and reason for entering the classical diagnostic flow for celiac disease. Non-eligible: volunteers who would not have been candidates for testing for celiac disease. CD compatible symptoms: volunteers who had been suffering from celiac disease related symptoms. CD-FDR: volunteers with a first degree relative with celiac disease. Autoimmune disorder: volunteers who suffered from an autoimmune disease.

In agreement with the ESPGHAN 2020 guidelines, 187/576 (32.46%) volunteers would have been candidates for testing for CD diagnosis because of the symptoms, signs and conditions declared on the clinical evaluation questionnaire (Figure 1). In contrast, 389/576 (67.53%) would most probably not have had confirmatory tests, because they do not fit into the ESPGHAN 2020 diagnostic flow (Figure 1) [14]. It is stated that these 187 would have been candidates for the CD tests because, at the time of screening inclusion, 151/187 (80.75%) volunteers had been suffering from at least one CD-related symptom for more than a month without an established cause; a total of 53/187 (28.34%) patients were aware of a CD-FDR, of whom 19/53 (34.85%) had CD-related symptoms; and 3 out of the 187 (3/187;1.60%) volunteers stated that they had been previously diagnosed with other autoimmune diseases: two with autoimmune thyroid disease, of which one had CD-compatible symptomatology, and one with type 1 diabetes (Figure 2).

### 3.3. Gluten Consumption Habits

The dietary gluten questionnaire, as a non-impartial measure of long-term gluten consumption, evaluates average dietary gluten exposure. In this study, of the 576 volunteers 481 (83.51%) volunteers completed the gluten consumption habits questionnaire, the 53 volunteers who confirmed to have a CD-FDR and 428 with none. According to it, the volunteers with CD-FDR were significantly less likely to consume gluten daily (*p* ≤ 0.05). Among the CD-FDRs, 48/53 (90.57%) declared eating gluten-made foods daily; among those who were not aware of having a CD-FDR, 412/428 (96.26%) stated they ate gluten-containing foods every day.

### 3.4. Anti-tTG-IgA Determination in Blood

Checking for the presence of anti-tTG-IgA and/or anti-EMA-IgA is usually where the diagnostic workup for CD starts [14,15]. All 576 children were screened for anti-tTG-IgA by using the Celiac Detect test. This anti-tTG-IgA test is a qualitative test; however, the following differentiations can be made regarding the test line intensity (Figure 2).

**Figure 3:**
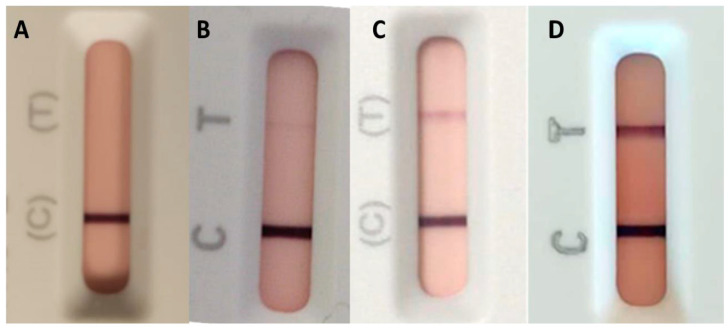
Anti-tTG-IgA lateral flow immunoassay test results according to the test line. (**A**) absent, (**B**) almost undetectable gray line, (**C**) faint or unsaturated red line, and (**D**) dark or saturated red line.

Volunteers who obtained a result with absence of the test line (Figure 3A) were considered negative (555/576). Eight (8/576) obtained an almost undetectable and unexpected gray test line (Figure 3B); any type of red-toned test lines, whether of weak (3/576) or strong (10/576) intensity (Figure 3C,D), were considered positive. In accordance with the instructions for use, any line that did not clearly have a red tone was considered as negative (Figure 3B). However, to ensure non-subjective validation of the test, volunteers with the presence of gray test lines were also followed for validation of the negative results. All volunteers who obtained a saturated positive result on the LFIA test (10/576) (Figure 3D) had anti-tTG-IgA levels >10× the ULN when measured by laboratory ELISA. Of the three volunteers who had an intermediate intensity result (3/576) (Figure 3C), one had >10× the ULN, and the remaining two had measurable anti-tTG-IgA levels, but below this threshold. Finally, the eight volunteers with a gray line showed unquantifiable anti-tTG-IgA seromarkers in the laboratory test, confirming that any non-clearly discernible, non-red-toned test lines should be considered as negative results. Therefore, the 13 volunteers with positive results were referred to a pediatric gastroenterologist for further examination and to verify the diagnostic characteristics of the test for this use.

### 3.5. Celiac Disease Diagnosis

Among the 13 volunteers who obtained positive results in the anti-tTG-IgA LFIA, 12 consented to the complementary tests necessary to confirm the diagnosis. The legal guardians of the thirteenth refused to carry out the additional tests for CD confirmation, arguing that the person in their care was in excellent health, despite explanations about the importance of the diagnosis and its subsequent follow-up from the doctors and the study promoters. This patient obtained a faint red test line in the LFIA. CD was confirmed in 11/12, as all of them had anti-tTG-IgA above the threshold of 10× ULN and tested positive for anti-EMA. The remaining patient, who obtained an unsaturated test line result in the screening test, presented anti-tTG-IgA titers of 60.8 U/mL, anti-EMA 1/20, and a biopsy classified as Marsh 0-I; thus, it will be followed up for a definitive CD diagnosis. Therefore, the LFIA tests showed a PPV of 91.67% (95% CI: 71.86–100%) for positive results with respect to CD presence, and the PPV obtained in this study was of 100% (95% CI: 95.45–100%) for saturated test line results.

The median age of the diagnosed volunteers was 9 years old (IQR: 4–11), showing no statistically significant differences from the average age of the overall group (*p* = 0.992). Five (5/313; 1.59%) were male and six (6/296; 2.29%) were female (*p* = 0.546). 

None of those diagnosed suffered from diseases associated with a higher risk for CD. Of the volunteers with a known CD-FDR, only one was aware of this at the outset, whilst three were defined as having CD-FDR during the study. Firstly, two siblings were diagnosed with CD in this study. Second, the mother of one of the volunteers was diagnosed in response to the diagnosis of her child. As a result, the CD rate with FDR increased from 1/53 (1.89%) to 4/56 (7.14%) in the study population.

Regarding symptoms, 5/11 of those diagnosed had CD-compatible symptoms dating from 3 months to more than 5 years before the screening. Iron-deficiency anemia was detected in three volunteers during the process of CD diagnosis. In general, most of the volunteers had symptoms related to CD for at least 1 year prior to testing (Table 1).

### 3.6. Evolution of the HR-QoL and Symptoms

Among the 11 volunteers diagnosed with CD in the screening, ten were followed to assess their HR-QoL evolution >6 months after the initiation of the GFD. The 11th participant could not be followed owing to communication issues. The symptoms and signs described in Table 1 disappeared or decreased in all volunteers. According to EQ-5D-Y answers, mobility problems disappeared for the volunteer who answered as having some, and the pain or discomfort disappeared or reduced for the four volunteers who showed at the beginning. One volunteer answered to be more worried and to have some problems for doing usual activities due to the GFD. The EQ-VAS score before the CD diagnosis was of 86.5 (95% CI: 74.9–98.1), and it increased to 93 (95% CI: 86.45–99.6) after starting the GFD (*p* = 0.355).

## 4. Discussion

The anti-tTG-IgA LFIA rapid test used in our study, from the 576 enrolled volunteers, identified 11 who were subsequently confirmed to have CD. Therefore, the CD prevalence of 1.91% (1:53; 95% CI: 1.08–2.74%) observed in this study was slightly higher than previously observed in Spanish children (1.41%; 1:71; 95% CI: 0.7–2.51%) [29]. Given that previously diagnosed patients with CD were excluded from this study, the anti-tTG-IgA LFIA functional parameters are likely similar to those offered by the common laboratory tests, which show a PPV of approximately 90% in pediatric children [7,15,42]. Moreover, the categorization of the results based on test line intensity could further stratify patients, as a saturated test line showed a PPV of 100% for anti-tTG-gA titers above 10× the ULN (95% CI: 95.45–100%) (essential criterion in some of the recent guidelines for CD diagnosis in the pediatric population, such as the ESPGHAN 2020 one) [14,15]. With further validation using a larger dataset, if the 100% PPV of saturated test lines for >10× anti-tTG-IgA ULN is maintained, it may be possible to remove the requirement to confirm such test results with laboratory-based ELISAs, resulting in a decrease in costs, risks, and refusals of diagnosis. In this scenario, the extra costs of validating the positive results with conventional laboratory anti-tTG-IgA testing would only be required for the 23.07% (3/13) of volunteers who presented with a non-saturated test line. In addition, there is a significant population of undiagnosed children who can be identified using anti-tTG-IgA LFIAs, as previously diagnosed patients with CD were excluded from this study. Differences in CD prevalence between the sexes and age groups were studied. As regards sex, no statistically significant differences were observed, although a 1:1.44 male:female diagnosis ratio was obtained, and a higher prevalence of symptoms was observed among females, in agreement with most studies [7,29,30]. In terms of age, neither were found statistically significant differences.

In the last decade, several LFIA tests have been developed for the detection of CD seromarkers. When comparing the PPVs obtained, the test used in this study is one of the most accurate, which would translate to a reduction in the number of people sent for additional diagnostic tests [7,43,44,45,46,47]. In studies performed with LFIA tests of other brands, the PPV differs between 8.23% to 96.8%, those detecting anti-deaminated gliadin peptide antibodies being the ones with lowest PPV rates [7,43,44,45,46,47]. To consider whether the Celiac Detect test added value in relation to existing diagnostic processes/protocols and to determine the extent to which a group might be diagnosed by screening tests, participants were divided into different groups according to the probability of diagnosis following the ESPGHAN 2020 criteria [14]. When all the volunteers were screened irrespective of their symptomatology and/or risk factors, CD was identified in 1.91% of the volunteers. When divided into different groups according to the probability of diagnosis following the ESPGHAN 2020 criteria, symptomatic patients with CD-FDR and/or an autoimmune a condition with a higher risk for CD could be considered to have the highest probability of diagnosis. However, only 20 volunteers in our study met these criteria, which may be insufficient to draw conclusions for this group [14,15,18]. Among children who were either symptomatic or had CD-FDRs (medium-high probability), CD was identified in 1.89–2.65%. In this study, no statistically significant differences were seen in CD prevalence between the average population and those who were known to be a CD-FDR, in spite of the existing scientific evidence stating that CD-FDR screenings yield approximately 7.5% of children being diagnosed with CD [24]. This could be due to higher rates of diagnosis among this group or the fact that family members of patients with CD may not consume enough gluten to raise antibody levels to detectable limits. Finally, 1.55% (95% CI: 0.62–2.49%) of the asymptomatic non-CD-FDR non-risk children were diagnosed with CD (Table 2) [14]. These data imply that in screening of global pediatric population, a comparable rate of diagnosis would be anticipated, irrespective of symptomology or the presence of CD-FDR (*p* = 0.526 [1/187], 0.569 [4/151], and 0.991 [1/53], respectively).

Concerning the symptoms, among the volunteers diagnosed with CD, 45.45% (5/11) had CD-compatible symptoms indicating their eligibility for CD testing as per the ESPGHAN 2020 [14]. All the symptomatic volunteers presented at least one CD-related symptom for over a year, and the appearance of other CD symptoms was not simultaneous, but they eventually appeared after the previous one (Table 1). Therefore, multiple volunteers may have had active CD for years before diagnosis, especially in volunteers aged >10-year-old, and may have suffered from clinical intestinal damage because of a delayed diagnosis. In contrast, 54.45% (6/11) did not belong to any risk group nor did they present symptoms associated with CD, and thus would have had a relatively low probability to enter the diagnostic stream based on the ESPGHAN 2020 criteria [14]. For asymptomatic patients without CD-FDRs, population screening would allow for the early diagnosis of many children and relatives who would otherwise not be identified. Indeed, the diagnosis of an asymptomatic volunteer triggered the diagnosis of their mother with CD-compatible symptoms. The availability of rapid anti-tTG-IgA tests in medical centers or the inclusion of such children in screening processes could speed up their diagnosis. These observations clearly demonstrate the limitations of prompt diagnosis based solely on active case-finding. 

To determine the short-term benefits of CD diagnosis in each group, the impact of the diagnosis was assessed by determining the changes in the HR-QoL scores obtained before and after >6 months of starting a GFD, which were determined through the answers obtained in the EuroQoL group EQ-5D-Y questionnaire [40,41]. Volunteers who reported physical symptoms confirmed a notable improvement in their HR-QoL after starting a GFD, while those who initially reported no symptoms noted neither an improvement nor a worsening in their health status, except for one participant who manifested more stress and had socializing difficulties due to treatment. This highlights both the short-term value of CD diagnosis for symptomatic CD and the difficulties associated with a GFD [8,36,38]. The HR-QoL study did not quantify the long-term health benefits associated with early CD detection. The fact that short-term improvements are visible only in symptomatic patients does not detract from the importance of identifying the disease in asymptomatic patients. Furthermore, the earlier the GFD is implemented, the better the adherence and GI mucosal health [31,32]. Therefore, such screenings could save considerable costs from other diagnostic tests, clinical visits, and associated patient deterioration, along with a reduction in patient discomfort [8,9,22,35,39,48]. 

In this study, it was not possible to determine the percentage of CD participants lost due to false negatives or due to IgA deficiency, as it was not possible to compare it with other tests and do more complementary tests. Nonetheless, it was interesting to check that those volunteers who had a CD-FDR were significantly less likely to consume gluten daily than those who had none (*p* < 0.05).

In summary, the simple rapid tests such as anti-tTG-IgA LFIAs could be of high clinical utility as they could help with the early identification of undiagnosed active CD cases. The time to obtain the result is less than 15 min, no extra equipment is required, and samples are obtained in situ in a less invasive way than other standard laboratory tests, thus eliminating the need for storage equipment and the risk of specimens being lost or misplaced. Children with diagnostic delays and those who did not meet the ESPGHAN 2020 diagnostic criteria obtained the highest benefit from LFIA-based screening for CD. Timely implementation of a GFD would help improve HR-QoL and would likely prevent long-term complications in all patients [9,22,23,26,39,48]. The costs of CD diagnosis would likely be reduced in the case of a saturated test line, given its high PPV for active CD. Furthermore, it could also potentially reduce the costs of undiagnosed CD if applied as a screening tool in general primary care, avoiding unnecessary referrals to gastroenterologists, reducing delays in diagnosis and long-term problems, and improving patients’ HR-QoL. Equally, these proceedings could easily be adopted in countries with more limited financial and logistics means and, therefore, with less access to health care resources and primary care centers. 

## Figures and Tables

**Table 1:** Clinical picture and risk factors of volunteers with celiac disease detected in the screening.

Volunteer		Onset of the Presence of Symptoms and Signs and Kinship with Celiac Disease Sufferers							
Sex	Age	Iron-Deficiency Anemia	Abdominal Pain	Diarrhoea or Chronic Constipation	Dermatitis Herpetiformis	Growth Stagnation	Irritability/Headache	Neuropathy	Celiac Disease Family
Male	4	n/a	n/a	n/a	n/a	n/a	n/a	n/a	Mother
Male	7	n/a	n/a	n/a	n/a	n/a	n/a	n/a	No
Male	10	n/a	>5 years	>5 years	n/a	n/a	n/a	>3 months ^1^	No
Male	11	n/a	n/a	n/a	n/a	n/a	n/a	n/a	Mother ^2^
Male	13	n/a	n/a	n/a	n/a	n/a	n/a	n/a	Sister ^2^
Female	2	unknown ^3^	1–5 years	>3 months	n/a	1–5 years	n/a	n/a	No
Female	3	unknown ^3^	1–5 years	n/a	n/a	1–5 years	n/a	n/a	No
Female	7	unknown ^3^	n/a	n/a	n/a	n/a	n/a	n/a	Brother ^2^
Female	9	n/a	n/a	n/a	n/a	n/a	n/a	n/a	No
Female	11	n/a	Unknown ^3^	>5 years	>5 years ^1^	1–5 years	>5 years	n/a	No
Female	14	n/a	n/a	n/a	n/a	n/a	n/a	n/a	No
Ratio of CD-diagnosed symptomatic volunteers		3/5	4/5	3/5	1/5	3/5	1/5	1/5	n/a
Ratio of CD-diagnosed volunteers		3/11	4/11	3/11	1/11	3/11	1/11	1/11	4/11

n/a: Not Applicable, due to the volunteer not showing this symptom. ^1^ Parents described symptoms whose characteristics could be associated with those of celiac disease. ^2^ Not aware of this relationship at the time of the screening. ^3^ Unknown: Not possible to date the onset of this symptom.

**Table 2:** Prevalence of undiagnosed celiac disease, positive predictive value of anti-tTG-IgA test, and screening cost, in line with the probability of meeting the requirements to be diagnosed as per the ESPGHAN 2020 criteria.

		Total	CD-FDR ^1^, Risk, and Symptomatic	Other Risk Factors	CD-FDR ^1^	Symptomatic	Non-CD-FDR ^2^, Non-Risk, and Asymptomatic
Prevalence	*n*	11/576	0/20	0/3	1/53	4/151	6/387
	%	1.91% (95%CI: 1.08–2.74%)	0%	0%	1.89% (95%CI: 0.00–4.95%)	2.65% (95%CI: 0.67–4.63%)	1.55% (95%CI: 0.62–2.49%)
PPV ^3^ (ratio)	All	11/12	n/a	n/a	1/1	4/4	6/8
	Saturated test line	10/10	n/a	n/a	1/1	3/3	6/6
PPV ^3^ (%)	All	91.67% (95%CI: 71.86–100%)	n/a	n/a	100% (95% CI: 50.00–100%)	100% (95% CI: 87.50–100%)	75.00% (95% CI: 38.74–100%)
	Saturated test line	100% (95%IC: 95.45–100%)	n/a	n/a	100% (95% CI: 50.00–100%)	100% (95% CI: 83.33–100%)	100% (95% CI: 91.67–100%)
Probability of diagnosis following the ESPGHAN ^4^ criteria		n/a	High	Medium-High	Medium-High	Medium-High	Low

n/a: Not Applicable. ^1^ CD-FDR, first-degree relatives of people with celiac disease. ^2^ Non-CD-FDR, non-first-degree relatives of people with celiac disease. ^3^ PPV, positive predictive value. ^4^ ESPGHAN, The European Society for Paediatric Gastroenterology Hepatology and Nutrition.

## Data Availability

The datasets generated during and/or analyzed during the current study are not publicly available, due to them containing data of under-age patients, but are available from the corresponding author on reasonable request.
